# Subcutaneous emphysema and ultrasound sonography

**DOI:** 10.1186/2052-0492-1-8

**Published:** 2013-11-27

**Authors:** Toshi Kubodera, Yushi U Adachi, Toshiyuki Hatano, Tadashi Ejima, Atsushi Numaguchi, Naoyuki Matsuda

**Affiliations:** Department of Emergency Medicine, Ogaki Municipal Hospital, Ogaki, Gifu, 503-8502 Japan; Department of Emergency Medicine, Nagoya University Hospital, 65 Tsurumai-cho, Showa-ku, Nagoya, Aichi, 466-8550 Japan; Department of Emergency and Critical Care Medicine, Nagoya University Graduate School of Medicine, 65 Tsurumai-cho, Showa-ku, Nagoya, Aichi, 466-8550 Japan

**Keywords:** Subcutaneous emphysema, Ultrasound sonography, Central venous catheterization

## Abstract

Subcutaneous emphysema is not a rare complication in intensive care unit patients. Recently, ultrasound guidance for central venous puncture is becoming popular; however, the information on imaging for subcutaneous emphysema is limited. We encountered a patient complicated with severe pneumomediastinum and subsequent subcutaneous emphysema. The catheter replacement was attempted, and we examined the visuality of cervical vessels using ultrasound sonography before the intervention. Internal jugular vein itself was observed despite of subcutaneously migrated air bubble; however, the range of ultrasound image was limited, and the relationship between the vessel and the adjacent tissue was unclear.

## Findings

Subcutaneous emphysema is not a rare complication in patients admitted to intensive care unit and received mechanical ventilation as well as with pneumomediastinum [1]. High positive end-expiratory pressure leading to excess airway strain would be one of the risk factors of the complications [2]. Patients requiring high airway pressure might be severely ill and need many medical interventions including central venous catheterization.

Recently, ultrasound guidance for central venous puncture is strongly recommended [3] and is sometimes required as a mandatory procedure [4] in hospitals. Ultrasound sonography is a useful and powerful tool for detecting the deep vein distinguished from the artery using the color Doppler imaging methods [5]. Moreover, sonography enables us to confirm the site of puncture for monitoring the spatial relationships between the venous and the needle during the puncture through the real-time imaging [6]. One of the important factors for complicating the ultrasound-guided central venous catheterization is subcutaneous emphysema as ultrasound barrier [7]. Absolute difference of acoustic impedance between the aqueous tissue and migrated air causing emphysema occludes the scattering of ultrasound signals and prevents from composing the image of deep body structures. Verniquet and Katel [7] reported the scanning image of the patient with subcutaneous emphysema; however, there is scarce information of the ultrasound images in a literature for the patient with subcutaneous emphysema.

**Figure 1:**
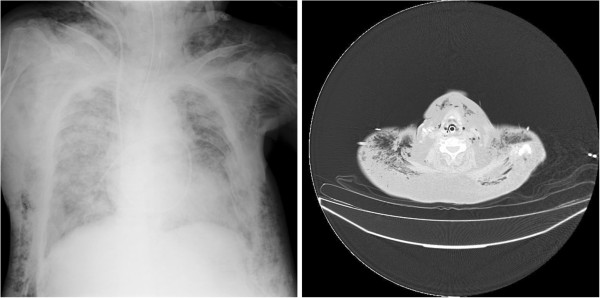
**The chest X-ray and CT findings of the patient.** Massive emphysema was observed in the neck, chest, and mediastinum.

We encountered a patient complicated with severe pneumomediastinum and subsequent subcutaneous emphysema in the intensive care unit (Figure 1). A 56-year-old female patient with adrenal insufficiency followed by severe sepsis and heart failure was required mechanical ventilation with high positive end-expiratory pressure for acute respiratory distress syndrome.

**Figure 2:**
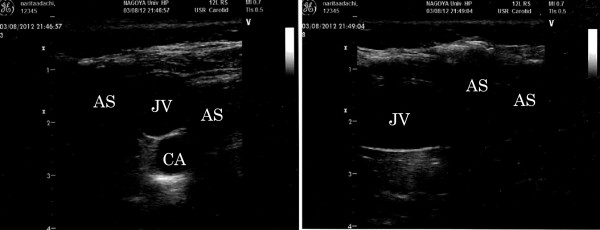
**Ultrasound images.** Left: The ultrasound image of the patient's neck in coronal view was demonstrated. The identification of both jugular vein and carotid artery was barely possible in spite of many subcutaneous ultrasound barriers by emphysema. Right: The ultrasound image in sagittal view was demonstrated. The jugular vein was feasibly observed; however, the carotid artery that is a relatively deep structure could not be identified. *JV* jugular vein, *CA* carotid artery, *AS* acoustic shadow by air.

Although the central venous catheter was already placed at admittance to the intensive care unit, replacement of the catheter was indispensable and planned for the long-term treatment. Repeated septic state and chronic infection also required the temporal removal of catheters. Thus, we examined the visuality of cervical vessels using ultrasound sonography before the intervention. In the case, internal jugular vein itself was observed despite of subcutaneously migrated air bubble; however, the range of ultrasound image was limited, and the relationships between the vessel and the adjacent tissue was unclear (Figure 2, left). Moreover, the ultrasound image of carotid artery was vague using long-axis in-plane approaches (Figure 2, right). The latter limitation could cause it difficult to detect an unexpected carotid puncture during central venous catheterization and following misplacement of thin guidewire into the artery [3, 6]. Subclavian approach was impossible because of the emphysema, and the access of femoral vein was considered as inappropriate because of the necessity of other blood accesses for renal replacement therapy. The catheter replacement was postponed until the clear imaging and safety access by ultrasound guidance was confirmed.

More and more information are gathered for reliable and safety catheterization using ultrasound guidance in the area of anesthesiology and intensive care medicine. We should continue to accumulate information of a plenty of images through daily clinical settings not only for normal subjects but also for complicated cases.

## Authors’ information

TK is a staff in the Emergency Department of Ogaki Municipal Hospital. He worked in the Department of Emergency and Critical Care Medicine, Nagoya University Graduate School of Medicine. YUA is an assistant professor in the Department of Emergency Medicine and the corresponding author of this letter. TH and TE are assistant professors of Emergency and Critical Care Medicine and consultants of ultrasound sonography. AN is an assistant professor of Emergency and Critical Care Medicine and a consultant of ultrasound echocardiography. NM is a professor and Chairman of Emergency and Critical Care Medicine.
